# Orexinergic neurons modulate stress coping responses in mice

**DOI:** 10.3389/fnmol.2023.1140672

**Published:** 2023-03-16

**Authors:** Jae Gon Kim, Ji Yun Ea, Bong-June Yoon

**Affiliations:** Department of Life Sciences, Korea University, Seoul, Republic of Korea

**Keywords:** major depressive disorder, anxiety, orexin, resilience, stress coping, chemogenetics, optogenetics

## Abstract

Stress is a critical precipitating factor for major depression. However, individual responses to the same stressor vary widely, possibly owing to individual variations in stress resilience. Nevertheless, the factors that determine stress susceptibility and resilience remain poorly understood. Orexin neurons have been implicated in the control of stress-induced arousal. Therefore, we investigated whether orexin-expressing neurons are involved in the regulation of stress resilience in male mice. We found that the level of c-fos expression was significantly different in susceptible versus resilient mice in the learned helplessness test (LHT). Furthermore, activating orexinergic neurons induced resilience in the susceptible group, and this resilience was also consistently observed in other behavioral tests. However, activating orexinergic neurons during the induction period (during inescapable stress exposure) did not affect stress resilience in the escape test. In addition, analyses using pathway-specific optic stimulation revealed that activating orexinergic projections to the medial part of the nucleus accumbens (NAc) alone mediated a decrease in anxiety but was not sufficient to induce resilience in the LHT. Collectively, our data suggest that orexinergic projections to multiple targets control diverse and flexible stress-related behaviors in response to various stressors.

## 1. Introduction

Stress is a clear precipitating factor for the development of psychiatric conditions such as major depressive disorder (MDD) and posttraumatic stress disorder (PTSD) (Lloyd, 1980). It has been estimated that up to 80% of MDD patients in community samples have had preceding stressful life events (Hammen, 2005). However, a negative life experience does not always result in psychopathological conditions. In fact, there is considerable variation in responses to stress in both human and animal models. While some people experience debilitating PTSD, others experience only acute and mild psychological disturbances or no symptoms at all after exposure to common stressors. It is thought that individual differences in stress vulnerability and resilience underlie this variation (Yehuda, 2004; Southwick and Charney, 2012). Stress resilience is generally defined as the ability to bounce back from adversity, trauma, or threats through efficient adaptation (Karatsoreos and McEwen, 2013). Understanding the mechanisms that subserve individual variations in stress resilience should help to improve current therapeutic strategies and aid the development of new treatment options for stress-related disorders.

Orexins are neuropeptides that are specifically expressed in neurons in the lateral hypothalamic area (LHA), perifornical area (PeF), and dorsomedial hypothalamus (DMH). Orexinergic neurons project to various brain regions that are implicated in the stress response or MDD, such as the paraventricular nucleus (PVN), central amygdala (CeA), ventral tegmental area (VTA), locus coeruleus (LC), and dorsal raphe (DR) nucleus (Peyron et al., 1998; Fadel and Deutch, 2002; Baldo et al., 2003; Carter et al., 2012). In fact, multiple studies have implicated orexinergic neurons in stress-related behaviors (Winsky-Sommerer et al., 2004; Johnson et al., 2010; Giardino and de Lecea, 2014; Bonnavion et al., 2015; Grafe and Bhatnagar, 2018). Furthermore, suicidal patients with MDD have been shown to exhibit reduced levels of orexin in the cerebrospinal fluid (Brundin et al., 2007a,b).

Herein, we investigated the role of the orexin system in stress susceptibility by using a learned helplessness paradigm. Our primary aim was to examine how the activity of orexinergic neurons might control stress-coping behavior after repeated stress exposures. We demonstrated that resilient mice showed higher c-fos expression in orexinergic neurons than susceptible mice after repeated stress exposure and that increasing the activity of orexinergic neurons in the LHA by chemogenetic activation led to a significant increase in active coping following the learned helplessness paradigm. Optogenetic stimulation of orexinergic neurons in the LHA also promoted active coping responses. In contrast, pathway-specific activation of orexinergic projections failed to produce changes in coping responses, but stimulation of orexinergic projections to the medial nucleus accumbens (NAcMed) resulted in anxiolysis in the novelty suppressed feeding (NSF) test. These data suggest that stress coping and anxiety after chronic stress might be regulated by fine orchestration of multiple orexinergic projections.

## 2. Materials and methods

### 2.1. Animals

Orexin-Cre transgenic mice, which were a kind gift from Dr. Takeshi Sakurai (University of Tsukuba), were maintained on the C57BL/6 J (SLC, Japan) background (Matsuki et al., 2009; Soya et al., 2017). All animals used in this study were male mice of 10–12 weeks old, which were maintained in a controlled environment under a 12:12-h light–dark cycle in the Gyerim Experimental Animal Resource Center (GEARC) of Korea University and provided free access to food and water. Mice that received cannula implantation were single-housed to avoid damage to the cannula. All experimental procedures involving animals were approved by the Institutional Animal Care and Use Committee of Korea University.

### 2.2. Viral vector preparation

All adeno-associated virus (AAV) vectors used in these experiments were constructed using the AAV Helper-Free System (cat# 240071, Agilent Technologies, CA, USA) as described previously (W. Kim et al., 2019).

The titers of the vectors used in our study were as follows: AAV-(DJ)-hSyn-double-floxed inverse open reading frame (DIO)-hM3Dq-mCherry, 9.1 × 10^8^ infectious units (IU)/μL; AAV-(DJ)-EF1α-DIO-ChR2-eYFP, 3.2 × 10^8^ IU/μL; AAV-retro-EF1α-DIO-ChR2-eYFP, 2.73 × 10^8^ IU/μL and AAV-retro-DIO-hm3dq-mCherry, 1.3 × 10^9^ IU/μL.

### 2.3. Stereotaxic microinjection

Adult male orexin-Cre transgenic mice were anesthetized by intraperitoneal (i.p.) injection of ketamine (115.36 mg/kg) and xylazine (23.32 mg/kg) and placed on a stereotaxic apparatus (David Kopf instrument).

For chemogenetic studies, we injected AAV(DJ)-hSyn-DIO-hM3Dq-mCherry into the bilateral LHA (0.27 μL/hemisphere; anteroposterior (AP), −1.3 mm; mediolateral (ML), ±1.1 mm; dorsoventral (DV), −5.1 mm) and AAV-retro-DIO-hM3Dq-mCherry into the bilateral NAcMed (AP, +1.7 mm; ML, ±1.5 mm; DV, −4.1 mm) or the VTA (AP, −3.4 mm; ML, ±0.5 mm; DV, −4.2 mm).

For the study involving optogenetic stimulation of orexinergic neurons, we injected AAV(DJ)-EF1α-DIO-ChR2-eYFP into the LHA (AP, −1.3 mm; ML, ±1.1 mm; DV, −5.1 mm) of orexin-Cre mice or AAV-retro-EF1α-DIO-ChR2-eYFP into the NAcMed (AP, +1.7 mm; ML, ±1.5 mm [10° angle]; DV, −4.1 mm) or the VTA (AP, −3.4 mm; ML, ±0.5 mm; DV, −4.2 mm). Then, we implanted optic ferrules (LC 1.25 mm OD, Multimode 304 Stainless Ferrules, Precision Fiber Products, Inc.) into the LHA bilaterally.

All virus injections were performed using a Nanoliter 2000 injector (World Precision Instruments). After each injection, the injector was kept in position for at least 3 min to avoid backflow. The injection site was examined in each mouse after completion of the experiments, and mice with off-target injections were excluded from the analyses.

### 2.4. Optogenetic stimulation

We applied a 473-nm light stimulus with a 15-ms pulse width at a frequency of 20 Hz, which was generated by a laser light source (MBL-FN-473-150 mW, CNI). Alternation between the 10-s ON phase and 10-s OFF phase was controlled by a pulse generator (model 575, BNC). The light intensity was measured at the tip of an optic fiber using a power meter (PM 100D, Thorlabs) on each day of the experiment and maintained at 5 mW for stimulation of LHA neuron somata. For releasing sufficient amount of orexin neuropeptide (van den Pol, 2012; Arrigoni and Saper, 2014), we applied photostimuli for 30 min (600 pulses/min, ×30 min). Optical stimulation was delivered to animals inside their home cages prior to behavioral tests.

### 2.5. Behavioral tests

All behavioral tests were performed under dim light in a soundproof behavioral chamber between Zeitgeber time (ZT)12 (lights off) and ZT16. For chemogenetic experiments, an i.p. administration of 1 mg/kg clozapine-N-oxide (CNO) (Tocris Bioscience, cat# 4936, Bristol, United Kingdom), a synthetic agonist of designer receptors exclusively activated by designer drugs (DREADDs), was administered 30 min prior to behavioral tests. For optogenetic experiments, animals were subjected to behavioral tests immediately after the completion of 30-min optical stimulation in their home cage. Mice behavior in the tests was video-recorded and the movies were analyzed with the video tracking software EthoVision XT 11.5 (Noldus). For details of each behavioral test, see supplementary methods.

### 2.6. Statistical analysis

All statistical analyses were performed using SPSS 24 (IBM). For variables with a nonparametric distribution, the Kruskal–Wallis test followed by the *post hoc* Mann–Whitney *U*-test (with Bonferroni correction) was used. One-way Analysis of variance (ANOVA) followed by the least significant difference (LSD) *post hoc* test was used for data that were normally distributed. Two-way repeated measure ANOVA followed by the LSD *post hoc* test or paired *t*-test was used to analyze data obtained by multiple observations. All data are presented as the mean ± standard error of the mean.

## 3. Results

### 3.1. Different stress coping strategies are associated with distinct levels of orexinergic neuron activation

To investigate whether hypothalamic orexinergic neurons are involved in stress responses, we first examined the effect of acute inescapable foot shock stress on the neuronal activity of orexinergic neurons. Acute stress induced an increase in the expression of c-fos within orexinergic neurons as previously shown (two-way ANOVA, *F*_stress (1,10)_ = 142.999, *p* < 0.001; Winsky-Sommerer et al., 2004; Johnson et al., 2010; Lee et al., 2016; Supplementary Figure 1).

**Figure 1:**
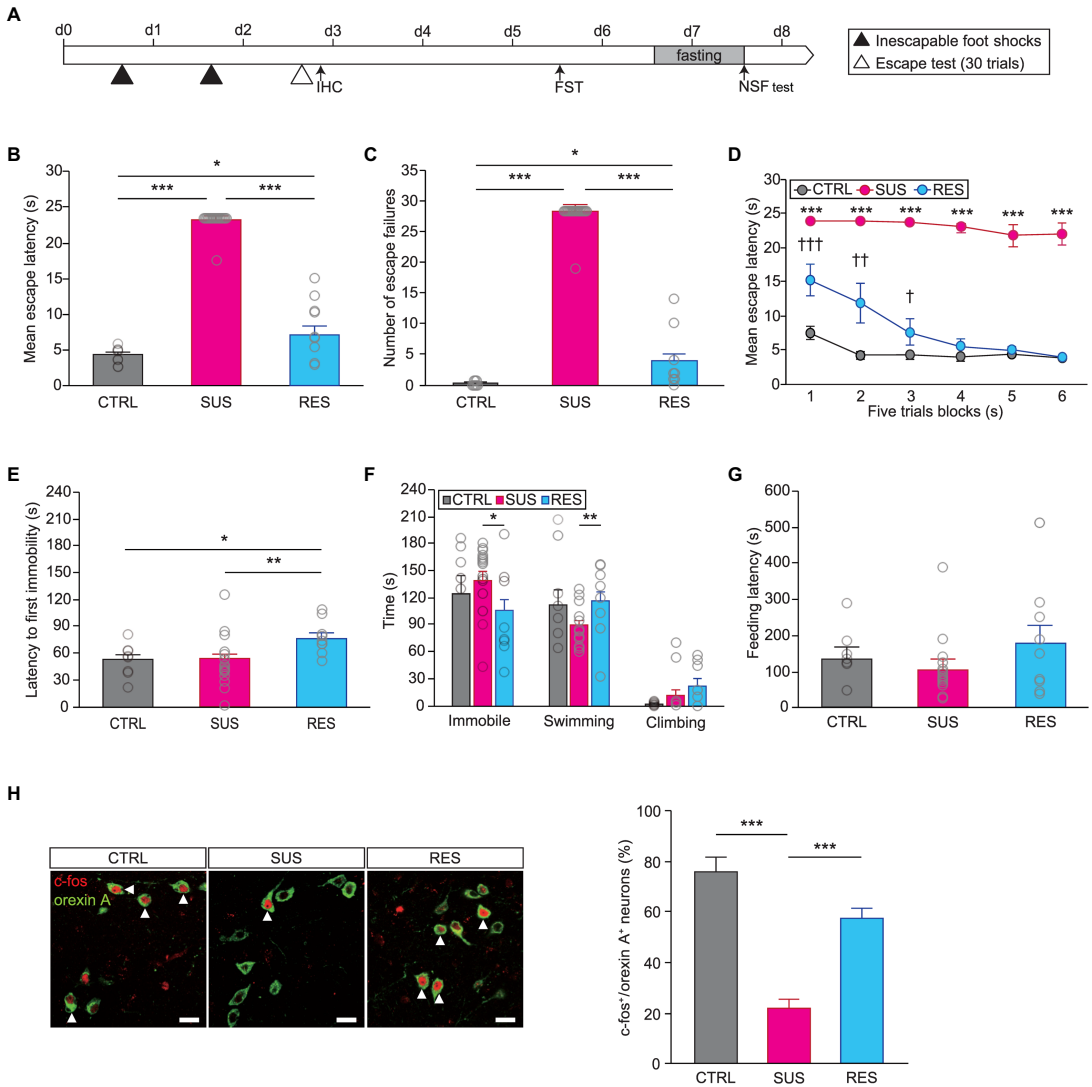
The activity of orexinergic neurons is significantly different between stress-resilient animals and susceptible animals. **(A)** Experimental schedule of the learned helplessness test (LHT) and other behavioral tests. A subset of animals was subjected to IHC after the first inescapable foot shock session or after the escape test [control (CTRL): *n* = 8, susceptible (SUS): *n* = 18, resilient (RES): *n* = 9]. **(B)** Susceptible mice displayed a significant delay in mean escape latency in the escape test (^*^p < 0.05, ^***^p < 0.001, Mann–Whitney U *post hoc* test). **(C)** Number of escape failures in the escape test (^*^p < 0.05, ^***^p < 0.001, Mann–Whitney U *post hoc* test). **(D)** Mean escape latency across blocks of five trials (^†^*p* < 0.05, ^††^*p* < 0.01, ^†††^*p* < 0.001, RES vs. CTRL, least significant difference (LSD) *post hoc* test; ^***^p < 0.001, SUS vs. CTRL, LSD *post hoc* test). **(E)** The latency to first immobility in the FST was significantly longer for the RES group (^*^p < 0.05, ^**^p < 0.01, LSD *post hoc* test). **(F)** Time spent immobile, swimming, or climbing in the FST (^*^p < 0.05, Mann–Whitney U *post hoc* test; ^**^p < 0.01, LSD *post hoc* test). **(G)** Latency to first feeding in the novelty suppressed feeding (NSF) test. **(H)** Immunofluorescence staining of orexinergic neurons with c-fos revealed a significant difference between groups. Representative images of c-fos expression in orexinergic neurons in the lateral hypothalamic area (LHA) in the three groups of mice are shown on the left (CTRL, *n* = 3; SUS, *n* = 5; RES, *n* = 4; scale bars indicate 20 μm). White arrowheads indicate cells double-labeled with c-fos (red) and orexin A (green). The quantitative results are shown in the graph on the right (^***^p < 0.001, LSD *post hoc* test). In all graphs, magenta, blue, and gray indicate the susceptible group, resilient group, and non-shocked control group, respectively.

Next, we performed the learned helplessness test (LHT) to assess the function of orexins after repeated exposure to unavoidable stress over which animals did not have control (Figure 1A). The learned helplessness paradigm has been widely used to generate animal models of MDD (Overmier and Seligman, 1967; Vollmayr and Henn, 2001) and has been shown to produce both susceptible and resilient phenotypes in mice (Chourbaji et al., 2005; Shin et al., 2015). We found that there were indeed individual differences in stress susceptibility, as measured by this protocol. We performed k-means clustering analysis using a python code based on their behavioral performance—the number of failures and escape latency—in the escape test to find two clusters and assigned each cluster to stress-susceptible group or stress-resilient group (Supplementary Figure 2; Shin et al., 2015). The susceptible group of mice (18 out of 27) displayed a significantly longer escape latency and a higher number of escape failures in the escape test than both the resilient group and nonshocked controls (escape latency, Kruskal–Wallis test, *H* = 29.903, *p* < 0.001; escape failures, Kruskal–Wallis test, *H* = 30.355, *p* < 0.001; Figures 1B,C). Analyzing the mean escape latency in five-trial blocks revealed that the susceptible group maintained a longer mean escape latency than the resilient group throughout the escape test. Interestingly, the resilient group displayed a longer escape latency than controls in the first two blocks but escaped as fast as the controls did in later blocks, indicating rapid recovery from a previous stressful experience (two-way repeated measures ANOVA, *F*_stress susceptibility (5,28)_ = 12.582, *p* < 0.001; *F*_stress_ susceptibility *x* trial blocks (10,56) = 4,284, *p* < 0.001; Figure 1D).

We investigated whether the observed difference in resilience in the LHT was correlated with behavioral responses in other tests. Subsequent behavioral tests were performed, and a difference in resilience was detected in the Forced swim test (FST). That is, the latency to first immobility was significantly longer for resilient mice than for susceptible mice (one-way ANOVA, latency to first immobility, *F*_(2,32)_ = 4.705, *p* < 0.05; Figure 1E). In addition, the time spent immobile was significantly shorter and the swimming time was significantly longer for resilient mice than for susceptible mice (Kruskal–Wallis test, immobility time, *H* = 6.826, *p* < 0.05; one-way ANOVA, swimming time, *F*_(2,32)_ = 4.985, *p* < 0.05; Figure 1F). Interestingly, resilient mice also differed from controls in FST, which indicates that resilience *per se* is qualitatively different from unstressed state. However, we did not find a significant difference between resilient mice and susceptible mice in performance in the NSF test (Figure 1G). This suggests that the experimental learned helplessness paradigm can affect behavior in various settings when an inescapable condition is presented, as in the FST, while anxiety-related behaviors, as measured in the NSF test, might be regulated by distinct or additional mechanisms. We then examined the c-fos expression in orexinergic neurons in these animals to determine the involvement of orexinergic neurons in stress resilience. A subset of animals was sacrificed 1 h after the escape test and processed for immunohistochemical analyses of c-fos expression. Interestingly, the susceptible group showed significantly lower c-fos expression in orexinergic neurons than the resilient group (two-way ANOVA, *F*_stress_ susceptibility (2,18) = 22.346, *p* < 0.001; Figure 1H), suggesting that the activity of orexinergic neurons might be critical for eliciting coping responses to stress after exposure to repeated uncontrollable stress.

### 3.2. Manipulation of the activation of orexinergic neurons in the test sessions but not in the induction sessions of the LHT affected stress coping responses

**Figure 2:**
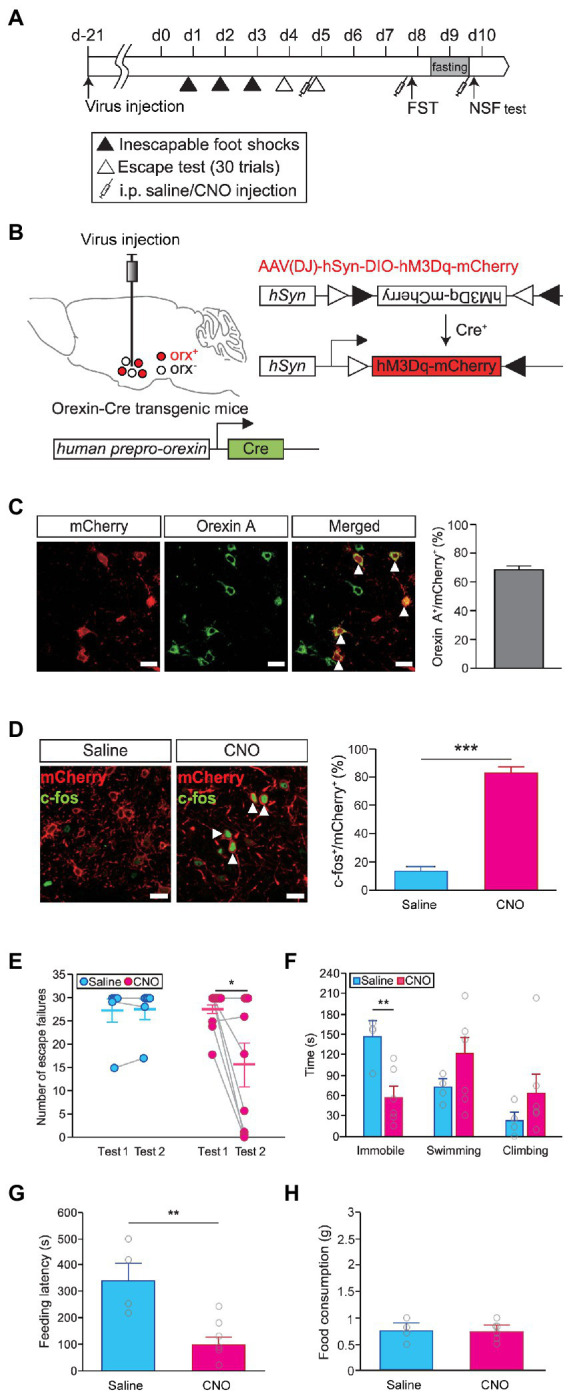
Chemogenetic activation of orexinergic neurons induces resilience. **(A)** Experimental schedule of chemogenetic activation of orexinergic neurons during stress-related behavioral tests [learned helplessness test (LHT): saline, *n* = 6; CNO, *n* = 9; FST and novelty suppressed feeding (NSF) test: saline, *n* = 4; CNO, *n* = 6]. **(B)** Schematic diagram of the induction of Cre-dependent hM3Dq expression in the lateral hypothalamic area (LHA) of orexin-Cre mice by viral vector injections. **(C)** Representative images showing colocalization of hM3Dq (red) and orexin A (green) in the hypothalamus (scale bars indicate 20 μm; left). The quantitative results are shown in the graph (right). White arrowheads indicate cells coexpressing hM3Dq (red) and orexin A (green). **(D)** Representative images showing c-fos expression (green) in hM3Dq-expressing cells (red) after saline or CNO injection (scale bars indicate 20 μm; left). The quantitative colocalization data are shown in the graph (^***^p < 0.001, independent *t*-test; right). White arrowheads indicate cells coexpressing mCherry (red) and c-fos (green). **(E)** Chemogenetic activation of orexinergic neurons in the second escape test induced a significant decrease in the number of escape failures (^*^p < 0.05, *post hoc* paired *t*-test). **(F)** Time spent immobile, swimming, or climbing in the FST upon chemogenetic activation (^**^p < 0.01, independent *t*-test). **(G)** The latency to the first feeding in the NSF test was also significantly reduced upon the activation of orexinergic neurons (^**^p < 0.01, independent *t*-test). **(H)** Total food consumption was not different between the CNO-treated and saline-treated groups in the NSF test.

We reasoned that if the level of orexinergic neuron activity was crucial for regulating stress resilience, it should be possible to change stress coping responses by modulating the activity of orexinergic neurons. We used a chemogenetic approach with DREADD to control the activity of orexinergic neurons *in vivo* (Figure 2A). An AAV vector harboring hM3Dq in the DIO configuration (AAV-DIO-hSyn-hM3Dq-mCherry) was injected into the hypothalamus of orexin-Cre transgenic mice to selectively express hM3Dq in orexinergic neurons (Figure 2B). We confirmed that hM3Dq colocalized with orexin A (Figure 2C) and that the activation of hM3Dq by CNO administration (1 mg/kg) resulted in a significant increase in neuronal activity, as measured by c-fos expression (independent *t*-test, saline vs. CNO, *t* = −12.450, *p* < 0.001; Figure 2D). To examine whether the activity of orexinergic neurons can causally affect stress resilience, we assessed helplessness upon concurrent activation of orexinergic neurons. We first categorized the animals into the resilient and susceptible groups based on basal behavioral performance in the escape test and then repeated the escape test for on the following day after hM3Dq activation, and then we used the susceptible mice for further analyses. Interestingly, the susceptible mice that had shown almost complete helplessness in the basal test displayed substantial resilience upon activation of orexinergic neurons, while saline-injected control animals maintained a helpless state (two-way repeated measures ANOVA, number of escape failures, *F*_test (1,13)_ = 5.128, *p* < 0.05; *F*_test × drug (1,13)_ = 5.427, *p* < 0.05; Figure 2E). In contrast, wild-type animals that received CNO injections without hM3Dq expression did not show a significant change in susceptibility (paired *t*-test; *t* = 1.0, *p* > 0.3) indicating that CNO alone did not affect behavior (Supplementary Figure 3). Consistently, the time spent in the immobile state in the FST was also significantly lower for CNO-treated animals than for saline-treated control animals (independent *t*-test, saline vs. CNO, *t* = 3.596, *p* < 0.01; Figure 2F). Apparently, the activation of orexinergic neurons facilitated an active coping effort to escape in both behavioral paradigms. Interestingly, we found that the activation of orexinergic neurons also altered behavioral responses in the NSF test. Animals that received CNO injection showed significantly earlier feeding attempts than saline-injected controls (independent *t*-test, feeding latency, saline vs. CNO, *t* = 3.572, *p* < 0.01; Figure 2G), while the amount of food consumption was not different between the CNO-injected animals and control animals (Figure 2H).

Next, we questioned whether the difference in behaviors between the susceptible and resilient groups was attributable to differential activity of orexinergic neurons during the induction sessions of the LHT, possibly due to genetic predisposition. To address this question, we modulated the activity of orexinergic neurons during the induction sessions and examined whether it influences the development of stress susceptibility. We activated orexinergic neurons in orexin-Cre mice injected with AAV-DIO-hSyn-hM3Dq-mCherry prior to inescapable foot shock exposures for three consecutive days in the induction period (Supplementary Figure 4A). However, activating orexinergic neurons during the induction period did not alter the observed susceptibility in the escape test (Supplementary Figures 4B,C). The behavior of the CNO-treated mice was indistinguishable from that of controls in the FST, as in the LHT (Supplementary Figure 4D). These findings suggest that the function of orexinergic neurons is important for the expression of stress-coping behavior, through which resilience is achieved, rather than for the development of stress tolerance during stress exposure.

### 3.3. Stress coping behaviors appear to result from the coordinated activity of orexinergic neuron network

Orexinergic neurons project to multiple targets in the brain (Harris and Aston-Jones, 2006). Since different areas in the brain are implicated in different functions, we explored the possibility that specific projections of orexinergic neurons to specific targets play a distinct role. To confirm the brain-wide projection pattern of orexinergic neurons, we examined the distribution of axons from orexinergic neurons in our orexin-Cre mice injected with AAV-CAG-DIO-tdTomato. We found strong orexinergic inputs to the nucleus accumbens shell (AcbSh) and multiple nuclei within the brain stem, including the VTA, DR, LC, and peduncular pontine tegmental nucleus (PPT). We also found lower-density innervations to the prefrontal cortex, paraventricular thalamus (PVT), and PVN of the hypothalamus. This innervation pattern of orexinergic neurons is consistent with what has been previously reported. Unlike what has been previously shown, however, we did not detect a significant distribution of orexinergic axons in the basolateral amygdala (BLA) or in the hippocampus (Peyron et al., 1998; Sakurai, 2007, 2014; Giardino and de Lecea, 2014; Supplementary Figure 5).

**Figure 3:**
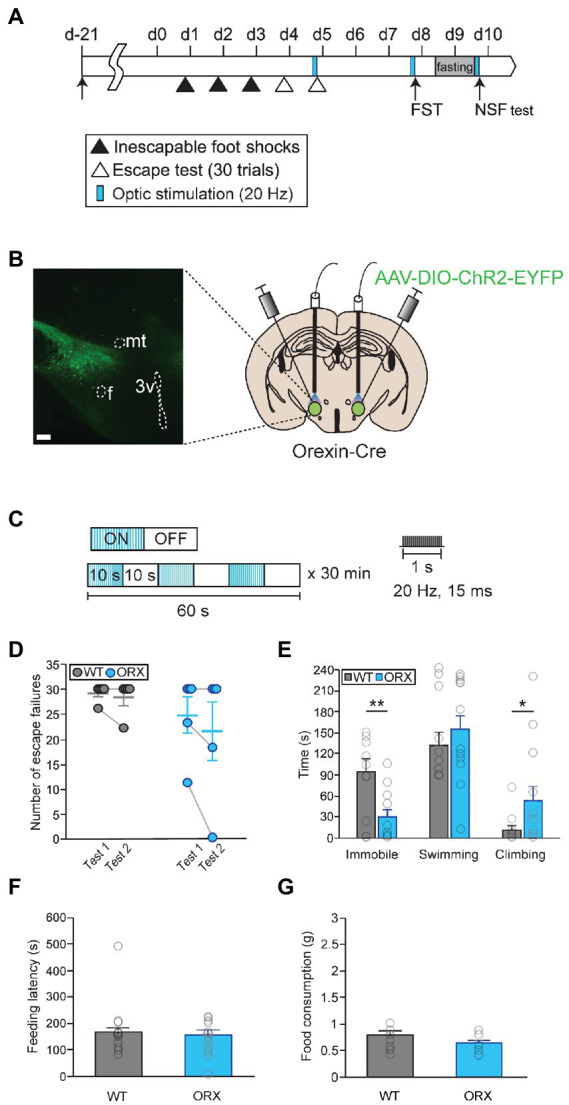
Optical photostimulation of lateral hypothalamic area (LHA) orexinergic neurons induces active coping behavior. **(A)** Experimental schedule of optogenetic activation of orexinergic neurons [WT, *n* = 11; orexinergic (ORX), *n* = 13]. **(B)** A representative image and diagram of the sites of virus injection and optical stimulation. f, fornix; 3v, third ventricle; mt, mammillothalamic tract (scale bar indicates 200 μm). **(C)** The optical stimulation paradigm used in this study. **(D)** Optogenetic activation of orexinergic neurons (blue) failed to induce a significant change in stress resilience in the learned helplessness test (LHT). **(E)** Optical stimulation of orexinergic neurons had a significant antidepressant-like effect in terms of immobility and climbing behavior in the FST (^**^p < 0.01, ^*^p < 0.05, Mann–Whitney U *post hoc* test). **(F, G)** Optical stimulation of orexinergic neurons did not induce a change in feeding latency or total food consumption in the novelty suppressed feeding (NSF) test.

To activate orexinergic neurons in a more temporally and spatially regulated manner, we used optogenetic approaches. First, we tried to confirm our results through chemogenetic activation of orexinergic neurons using an optogenetic approach (Figures 3A,B). Prolonged optical stimulations (20 Hz with a 10-s pulse and a 10-s interpulse interval for 30 min) were delivered prior to each behavioral test to ensure that peptidergic terminals were activated (Figure 3C; Bonnavion et al., 2015). Overall locomotor behavior during optical stimulation did not differ in orexin-Cre mice and in wild-type control mice, implying that orexinergic neuron activation did not change general motor activity (Supplementary Figure 6). Surprisingly, stimulating orexinergic neurons prior to the LHT and NSF test failed to induce significant changes in stress responses (Figures 3D,F,G). In contrast, it led to a significant decrease in passive coping and an increase in active coping behavior in the FST (Mann–Whitney *U*-test, immobility time, *U* = 27.5, *p* < 0.01; Mann–Whitney *U*-test, climbing, *U* = 112.5, *p* < 0.05; Figure 3E).

**Figure 4:**
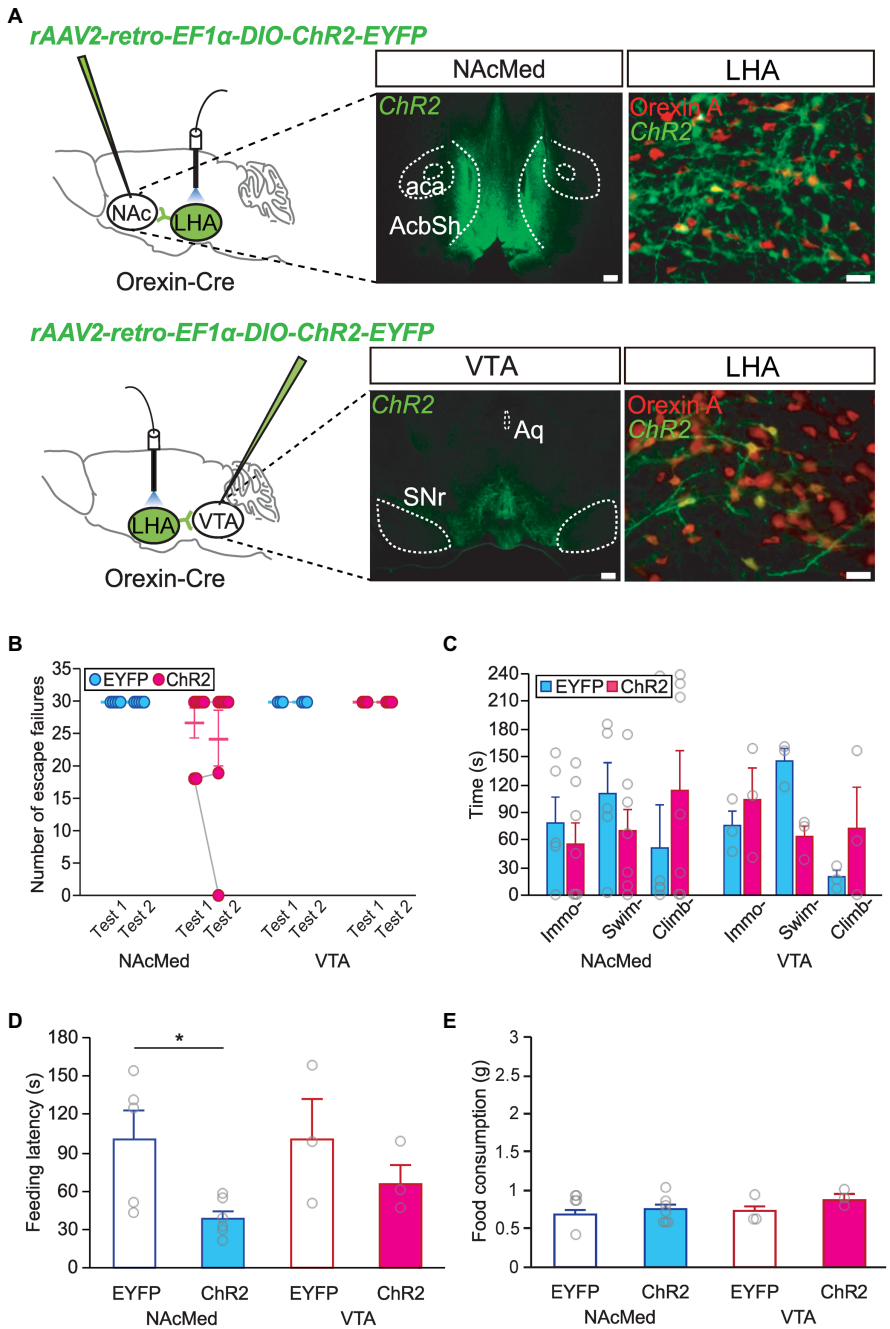
Orexinergic inputs to the NAcMed mediate food-seeking behavior in the novelty suppressed feeding (NSF) test. **(A)** Representative images and diagrams of the sites of virus injection [NAcMed or ventral tegmental area (VTA), scale bar indicates 200 μm] and optical stimulation [lateral hypothalamic area (LHA), scale bar indicates 20 μm]. AcbSh, nucleus accumbens shell; aca, anterior commissure; Aq, aqueduct; SNr, substantia nigra pars reticulata. **(B)** Optogenetic activation of orexinergic neurons projecting to the NAcMed or the VTA failed to induce a significant change in stress resilience in the learned helplessness test (LHT) (NAcMed, EYFP *n* = 5, ChR2 *n* = 7; VTA, EYFP *n* = 3, ChR2 *n* = 3). **(C)** Optogenetic activation of orexinergic neurons projecting to the NAcMed or the VTA failed to induce a significant behavioral change in the FST. **(D)** Activating orexinergic neurons projecting to the NAcMed induced a significant reduction in feeding latency in the NSF test (^*^p < 0.05, independent *t*-test). **(E)** Total food consumption in the NSF test was not different among groups.

As the mesolimbic dopamine system has been proposed to be involved in major depression and stress responses (Krishnan et al., 2007; Vialou et al., 2010; Cabib and Puglisi-Allegra, 2012), we investigated whether a specific orexinergic circuit is responsible for resilient behavior after stress. To activate orexinergic neurons making specific projections, orexin-Cre mice were injected with a retrograde AAV encoding ChR2 (AAV-retro-DIO-ChR2-EYFP) into each target site for optogenetic experiments (Figure 4A). We found that activating nucleus accumbens (NAc)-projecting or VTA-projecting orexinergic neurons alone did not affect helplessness behavior in the LHT (Figure 4B) or FST (Figure 4C). However, optic stimulation of the LHA-to-NAcMed orexinergic circuit resulted in decreased anxiety behavior. The latency to feed in the NSF test was significantly shorter in animals in which orexinergic neurons projecting to the NAcMed had been activated than in the EYFP-expressing control animals (independent *t*-test, NAcMed, EYFP vs. ChR2, *t* = 2.714, *p* < 0.05; Figures 4D,E).

## 4. Discussion

Stress responses are physiological and psychological adaptations to stressors that are perceived as threatening or dangerous. These responses engage and affect multiple neural networks, such as the autonomic nervous system, hypothalamic-pituitary-adrenal (HPA) axis, and motivational network. These brain network functions control and coordinate physiological and behavioral adaptation to maintain homeostasis and organismal well-being. However, when these responses are dysregulated or overused, they can lead to maladaptation that may hamper individual health and survival (McEwen and Gianaros, 2011). Proper adaptive responses require fine coordination of these hormonal and neural networks to produce effective coping and resilience. The present work examined whether orexinergic neurons in the hypothalamus participate in adaptive stress responses and affect coping responses to stressors.

We found that orexinergic neurons were strongly activated by acute stress and that resilient animals demonstrated a significantly higher level of orexinergic neuron activation after chronic stress than susceptible animals. This suggests that orexinergic neurons are critically involved in stress responses. Furthermore, we showed that chemogenetic activation of orexinergic neurons in stress-susceptible mice induced an increase in active coping behavior, indicating that the orexin system is a key regulator of stress resilience. Unlike chemogenetic activation, optogenetic stimulation of orexinergic neurons did not fully reproduce the resilient phenotype in the LHT, although it led to a decrease and an increase in passive coping and active coping, respectively, in the FST. This difference might have been due to the timing of activation during the test. For technical reasons, we applied optogenetic stimulation prior to the test but not during the test. While it has been shown that the optogenetic activation of hypothalamic neurons elicit behaviors that outlast stimulation due to persistent population activity (Kennedy et al., 2020), it is not clear whether orexinergic neurons do possess comparable property and to what extent optical stimulations applied prior to the tests affect behavior. In addition, optogenetic stimulation has its limitation in that light penetrance and spread is restricted. This might have been the source of variability in our optogenetic experiments and requires further investigation.

Orexinergic neurons have multiple projection targets, including the PVN, LC, NAc, and VTA, which are directly or indirectly implicated in stress responses. This implies that orexinergic neurons are strategically positioned to orchestrate stress responses and coping behavior in various contexts. For example, acute corticosterone responses through the HPA axis can be stimulated by orexinergic inputs to PVN neurons that produce corticotropin-releasing factor (Kuru et al., 2000; Sakamoto et al., 2004). This initial stress response signals the body to get ready physiologically and metabolically for an incoming threat. When a threat is assessed to be serious enough to require further coping actions, orexinergic neurons might function to guide active or passive coping responses through stimulating the motivational network, including the mesolimbic pathway or hypothalamic motivational system, or the arousal network in the brain stem (Narita et al., 2006; Shin et al., 2009; Sears et al., 2013; Kosse et al., 2017; Blomeley et al., 2018). Resilience can be achieved through appropriate coping responses, such as escaping from the source of danger, which are likely to be mediated by the coordinated activity of multiple neural circuits. Our findings demonstrated that the activation of orexinergic neurons in a stressful situation can induce animals to exhibit active coping. Although prior optical stimulation of a subset of orexinergic neurons projecting to the NAcMed or VTA failed to produce active coping behavior, approaching food in the NSF test was prompted by the optogenetic stimulation of orexinergic projections to the NAcMed. A similar trend was observed when we activated the specific projections of orexinergic neurons using DREADD, although it did not reach a statistical significance (Supplementary Figure 7). There is no imminent threat in the NSF test, and behavior is instead governed by the motivation to seek food and assessment of the remote possibility of danger or anxiety. Considering that the NAc is a major target area for dopaminergic inputs, the delicate interplay between orexinergic and dopaminergic inputs might critically control the function of the NAcMed, which determines behavioral responses to stress and motivation. Our data are somewhat contradictory to those presented in a previous report showing that orexinergic input to the NAc enhances risk-avoidance behavior (Blomeley et al., 2018). While it is currently not clear how to reconcile these different results, it is conceivable that orexinergic neurons might regulate active behavior both positively and negatively depending on the level of stress.

In conclusion, we found that orexinergic neurons play a critical role in controlling stress responses. Particularly, active coping responses are promoted by increasing overall orexinergic activity but not by a subset of neurons projecting to the mesolimbic pathway. These findings suggest that active coping responses are mediated by the activation of multiple circuits that are coordinated by orexinergic inputs. In contrast, food seeking behavior under possible risk conditions is sufficiently promoted by NAcMed-projecting orexinergic neurons. Our findings indicate that fine tuning of orexinergic projections to multiple targets is critical for determining the appropriate action strategy and could help to establish a new therapeutic paradigm aimed at increasing resilience in the content of psychiatric diseases.

## Data availability statement

The original contributions presented in the study are included in the article/Supplementary material, further inquiries can be directed to the corresponding author.

## Ethics statement

The animal study was reviewed and approved by Korea University Institutional Animal Care and Use Committee.

## Author contributions

BY conceived the study. BY, JK, and JE designed the analyses and wrote the manuscript. JK and JE performed material preparation, data collection and analyses. All authors contributed to the article and approved the submitted version.

## Funding

This study was supported by the Basic Science Research Program through the National Research Foundation of Korea (NRF) funded by the Ministry of Science, ICT, and Future Planning of the Republic of Korea (nos. NRF-2014R1A2A1A11053247 and NRF-2021R1I1A2052948) and a Korea University research grant.

## Conflict of interest

The authors declare that the research was conducted in the absence of any commercial or financial relationships that could be construed as a potential conflict of interest.

## Publisher’s note

All claims expressed in this article are solely those of the authors and do not necessarily represent those of their affiliated organizations, or those of the publisher, the editors and the reviewers. Any product that may be evaluated in this article, or claim that may be made by its manufacturer, is not guaranteed or endorsed by the publisher.
