# Supplementary material for: Orexinergic neurons modulate stress coping responses in mice
Source: Front Mol Neurosci. 2023 Mar 16;16:1140672. doi: 10.3389/fnmol.2023.1140672 (PMC10061830; doi:10.3389/fnmol.2023.1140672)
Supplement: Supplementary file 1 [file Data_Sheet_2.docx]

**Supplementary materials**

**Orexinergic neurons modulate stress coping responses in mice**

Jae Gon Kim, Ji Yun Ea, Bong-June Yoon^*^

*** Correspondence:** Bong-June Yoon: bjyoon69@korea.ac.kr

**Supplementary methods**

## Learned helpless test (LHT)

During the induction period, mice were placed on one side of a two-chamber shuttle box (42 cm × 16 cm × 22 cm) and randomly administered inescapable foot shocks (0.3 mA, 100 times/day) produced by a shock generator (Standalone Aversive Stimulator, ENV-414S, Med Associates Inc.) for 2–3 days. The intershock interval varied randomly from 5–60 s, and the duration of each shock pulse was 5 s. Animals were subjected to the escape test, which consisted of 30 trials with an intertrial interval of 30–60 s, on the following day. In the initial five trials, the gate between the two chambers was opened at the onset of shock delivery without delay. In the next 25 trials, there was a 2-s delay between shock onset and gate opening. A trial was terminated when the animal escaped or failed to escape within 30 s. The latency to escape for each trial and the number of escape failures in the 30 trials were measured. Based on the result of k-means clustering analysis using escape latency and the number of escape failures in the first escape test, the animals were divided into the susceptible and resilient groups. We subsequently assigned the animals to either susceptible group or resilient group based on the behavioral cutoffs obtained from the clustering analysis (escape latency: 16.3 s, escape failures: 16).

## Forced swimming test (FST)

The FST was performed as previously described (J. W. Kim et al., 2013) and was conducted during the dark cycle. Briefly, animals were placed into an acrylic cylinder (44.5cm height, 20cm diameter) filled with 5 liters of distilled water at 23°C and subjected to a forced swim session for 6 minutes. The immobile, swimming, and climbing phases were determined from video analysis based on paw movements and posture. Less than 2 minor strokes per second was assessed as immobile phase and major strokes of all four paws with upright posture per second was assessed as climbing phase. First 2 minutes of a FST session was excluded from analysis. Analyses were performed by trained personnel who were blind to the test conditions.

## Novelty suppressed feeding (NSF) test

Mice that had been deprived of food for 24 h were placed in a corner of an open field arena (30 cm×30 cm×45 cm), which was filled with 2-cm–thick bedding. Food pellets were placed in the center of the arena on white filter paper (15-cm diameter) that was illuminated by a focused LED light (450 lux). The latency to the first continuous feeding (lasting 5 s) during the 10-min test period was measured. The first feeding was stopped immediately after 5 s and the animal was returned to its home cage, which contained pre-weighed food pellets, and allowed to access food freely for 30 min. The total amount of food consumed was measured. If an animal failed to make a feeding attempt within 10 min, the test was terminated by returning the mouse to its home cage for free food consumption.

## Immunohistochemistry (IHC)

Immunohistochemical studies were performed as previously described (Young Lee et al., 2020). Briefly, mice were deeply anesthetized with a mixture of ketamine (150 mg/kg) and xylazine (15 mg/kg). The brain was extracted after fixation by cardiac perfusion with chilled 4% paraformaldehyde in phosphate buffered saline (PBS) and post-fixed for 4 h. Samples were then stored in 30% sucrose solution at 4 °C until they sink. Brain sections were made into 40-µm-thick slices using a cryostat (Leica CM1950, LEICA) and washed in 0.1 M PBS. Samples were incubated with blocking buffer (3% bovine serum albumin, 0.5% Triton X-100 in 0.1 M PBS) for 1 h, then incubated with a primary antibody in blocking buffer overnight at 4 °C and subsequently with a secondary antibody in blocking buffer for 1 h at room temperature. Samples were then mounted with a mounting medium (Vectashield, Vector laboratories). The following antibodies were used in this study: rabbit antiserum against orexin-A (1:300; cat# Y450, Yanaihara Institute Inc.), goat anti-c-fos (1:200; cat# sc-52-G, Santa Cruz Biotechnology), Alexa Fluor 488-conjugated donkey anti-rabbit IgG (1:400; cat# A-21206, Thermo Fisher Scientific), and Alexa Fluor 594-conjugated donkey anti-goat IgG (1:400; cat# A-11058, Thermo Fisher Scientific).

**Reference**

Kim, J. W., Ahn, H. S., Baik, J. H., & Yoon, B. J. (2013). Administration of clomipramine to neonatal mice alters stress response behavior and serotonergic gene expressions in adult mice. Journal of Psychopharmacology, 27(2), 171-180.

Lee, Y., Han, N.-E., Kim, W., Kim, J. G., Lee, I. B., Choi, S. J., et al. (2020). Dynamic Changes in the Bridging Collaterals of the Basal Ganglia Circuitry Control Stress-Related Behaviors in Mice. Molecules and cells, 43(4), 360. doi: 10.14348/molcells.2019.0279.

**Supplementary figures**

**Supplementary Figure 1.** **Acute foot shock stress induces a significant increase in the activity of hypothalamic orexinergic neurons.** (A) Representative images of c-fos expression (red) in orexinergic neurons (green) after foot shock exposure. Hypothalamic orexinergic neurons in both the dorsomedial hypothalamus (DMH) and lateral hypothalamic area (LHA) showed an increase in c-fos expression (control (CTRL), n=4; foot-shocked (FS), n=3; scale bar indicates 20 µm). White arrowheads indicate cells double-labeled with c-fos (red) and orexin A (green). (B) Quantification of c-fos expression in orexinergic neurons after foot shock in hypothalamic regions are shown in the graph (***p<0.001, LSD post hoc test).

**Supplementary Figure 2. Mice that underwent LHT were sorted by k-means clustering analysis using a python code (n=27).** Clustering was performed using two variables, escape latency and escape failures. We determined the arithmetic means of two nearby animals from each cluster and used those as cutoffs in subsequent experiments. The cutoffs are displayed as dashed line (Escape latency: 16.3 s, Number of failures: 16).

**
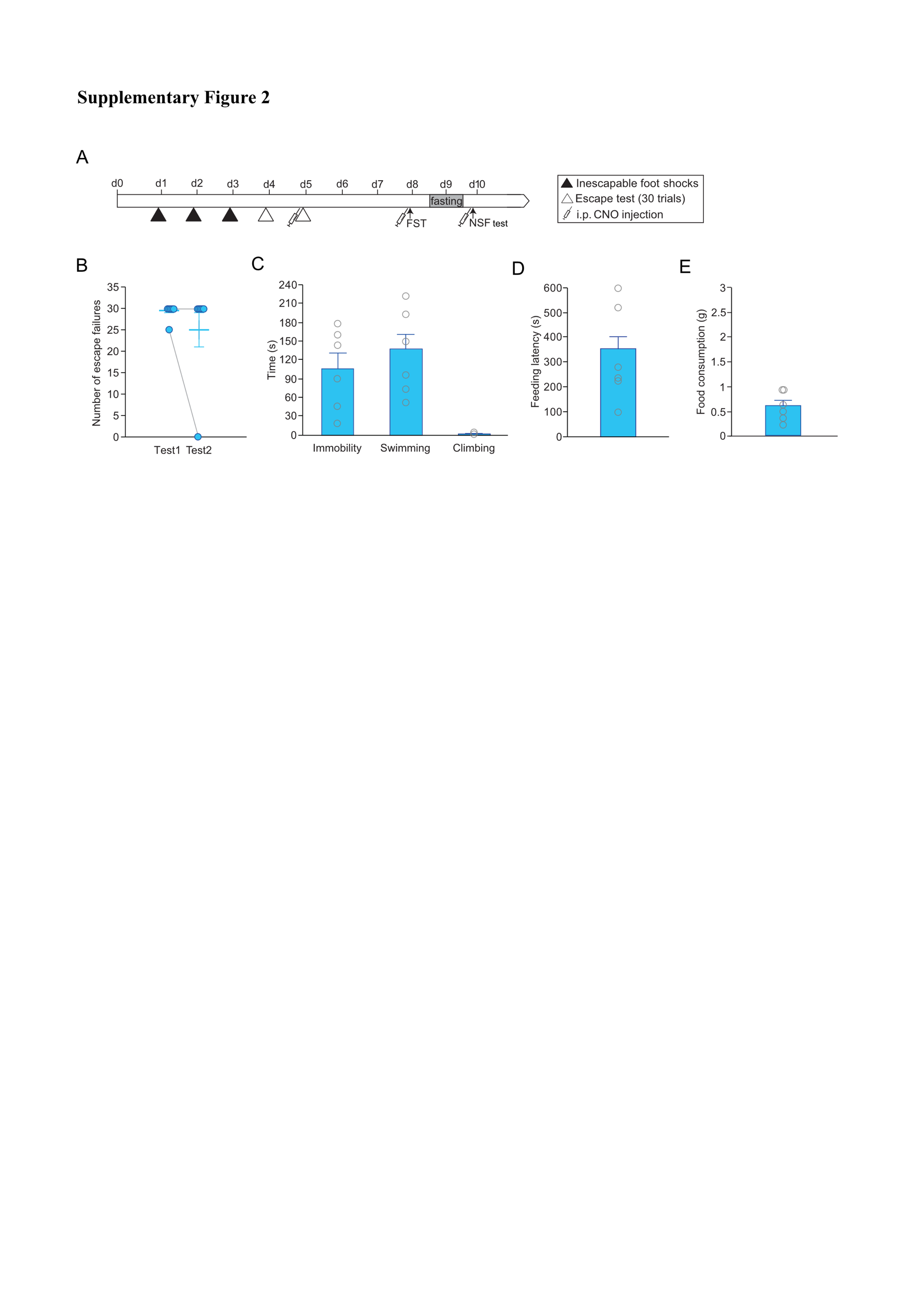
**

**Supplementary Figure 3. CNO administration without DREADD expression does not affect stress susceptibility.** (A) Experimental schedule of stress-related behavioral tests with CNO administrations (n=6). (B) CNO administration per se did not significantly change the number of escape failures in LHT. (C-E) Behavioral measurements in FST (C) and NSF test (D, E) after CNO administration.


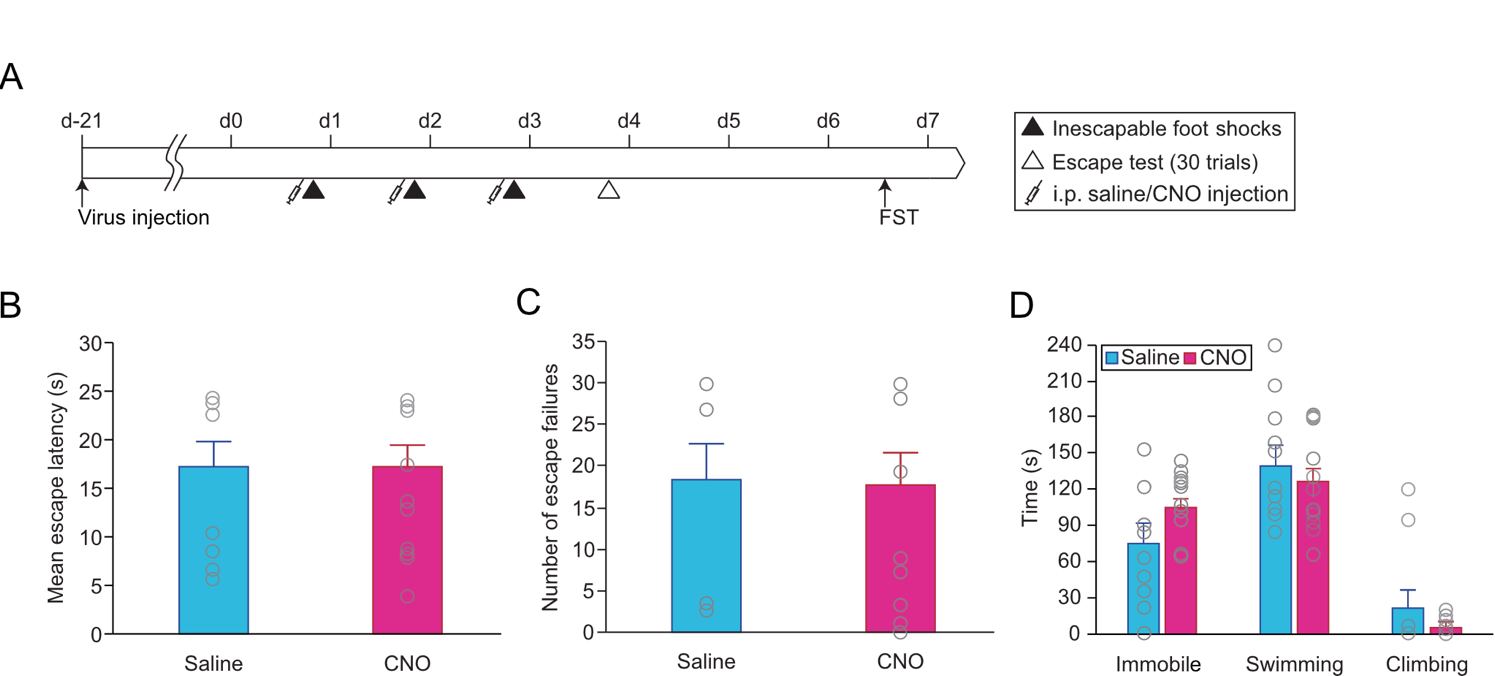


**Supplementary Figure 4. Chemogenetic activation of orexinergic neurons during inescapable stress exposure does not affect stress susceptibility.** (A) Experimental schedule of chemogenetic activation of orexinergic neurons during the induction sessions (saline, n=10; CNO, n=12). (B) The mean escape latency in the escape test of the LHT, (C) number of escape failures in the escape test of the LHT, and (D) time spent immobile, swimming, or climbing in the FST did not change after chemogenetic activation during inescapable foot shock exposure.


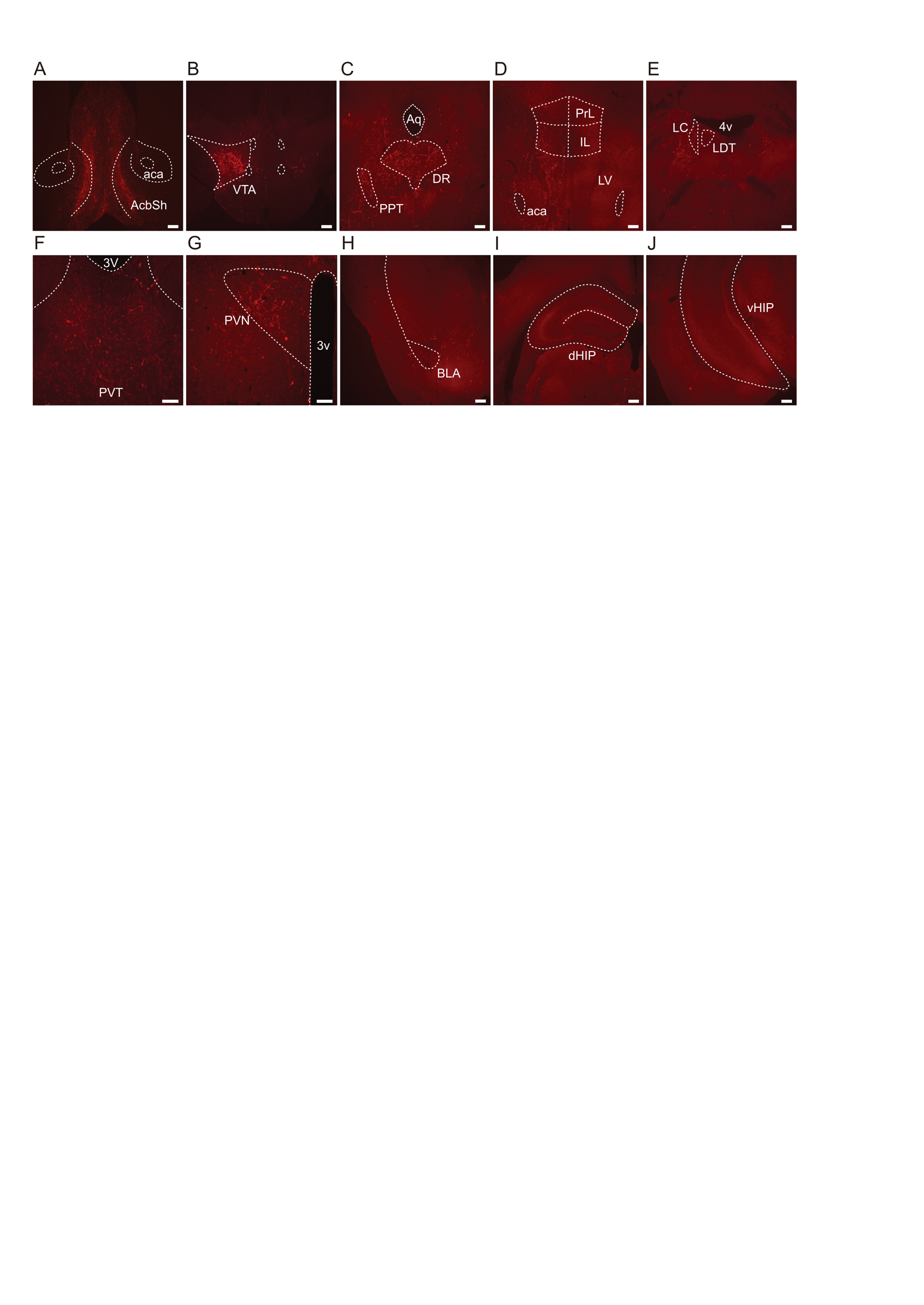


**Supplementary Figure 5. Distribution of the axons of orexinergic neurons in various brain areas.** AAV-CAG-DIO-tdTomato was injected into the LHA of orexin-Cre mice, and the axonal distribution of neurons expressing tdTomato was examined. (A, B) Orexinergic neurons sent dense projections to the NAcMed and VTA. (C-G) Medium to high fluorescence signals were detected in midbrain nuclei, the prefrontal cortex, a thalamic nucleus and a hypothalamic nucleus. (H-J) No signal was detected in the amygdala or hippocampus. aca, anterior commissure; 3v, third ventricle; PrL, prelimbic cortex; IL, infralimbic cortex; LV, lateral ventricle; 4v, fourth ventricle; LDT, lateral dorsal tegmental; dHIP, dorsal hippocampus; vHIP, ventral hippocampus. The scale bar indicates 20 µm in (F, G) and 200 µm in (A-E) and (H-J).

**
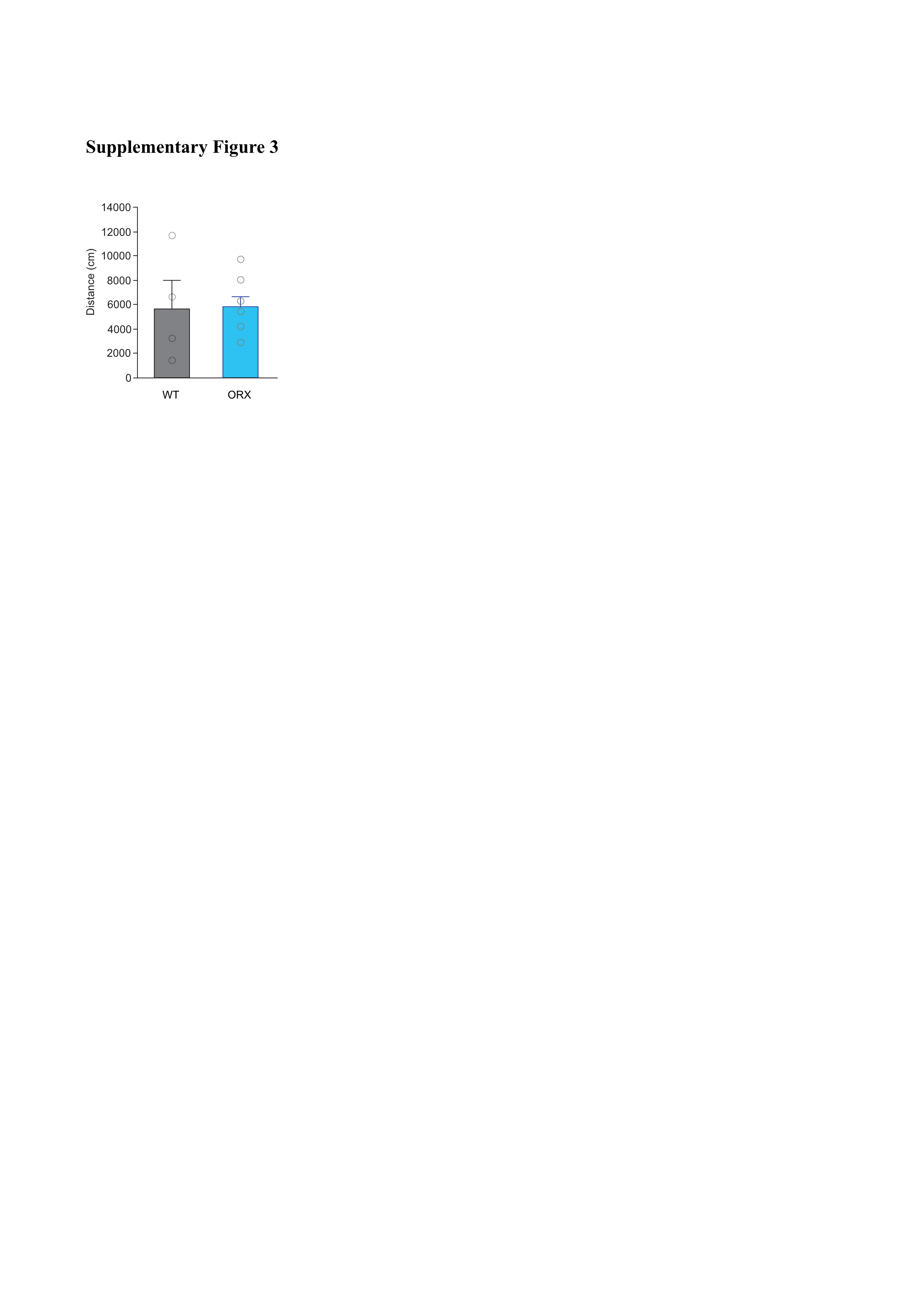
**

**Supplementary Figure 6. Locomotor activity measured from wildtype mice (WT) and orexin-Cre mice (ORX) during optical stimulation in their home cage.** WT and ORX displayed comparable motor activity indicating that stimulating orexinergic neurons did not affect general motor activity (WT n=4, ORX n=6).

**
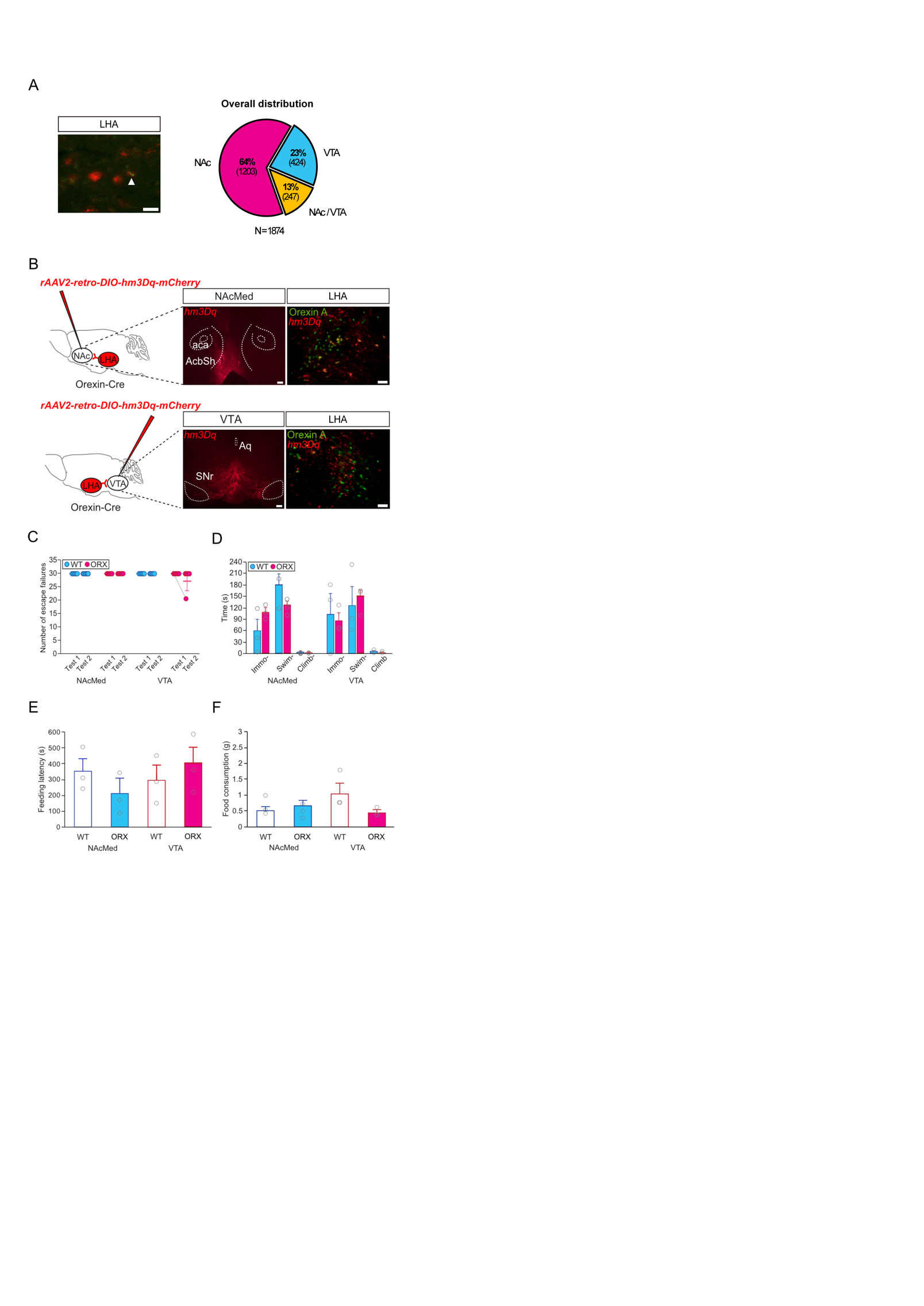
**

**Supplementary Figure 7. Projection-specific activation of orexinergic neurons using chemogenetics.** (A) Projections from the LHA to NAcMed and VTA were confirmed by injecting retrobead red into the NAcMed and retrobead green into the VTA. A white arrowhead indicates neuron projecting to both NAcMed and VTA (left). Representative image and quantified results in a pie chart are shown (right). (B) Diagrams showing experimental schemes. Retrograding AAV expressing hM3Dq was injected into the projection target sites of orexinergic neurons. Representative images showing the sites of virus injection (NAcMed or VTA, scale bar indicates 200 µm) and colocalization of hM3Dq (red) and orexin A (green) in the LHA (scale bar indicates 20 µm) are shown on the right. AcbSh, nucleus accumbens shell; aca, anterior commissure; Aq, aqueduct; SNr, substantia nigra pars reticulata. (C) Chemogenetic activation of orexinergic neurons projecting to the NAcMed or the VTA failed to induce a significant change in stress resilience in the LHT (NAcMed, WT n=3, ORX n=3; VTA, WT n=3, ORX n=3). (D-F) Behavioral measurements after chemogenetic activation of orexinergic neurons projecting to the NAcMed or the VTA in FST (D) and NSF test (E, F).
